# Supplementary material for: Effectiveness and Safety of Ayurvedic Medicines in Type 2 Diabetes Mellitus Management: A Systematic Review and Meta-Analysis
Source: Front Pharmacol. 2022 Jun 8;13:821810. doi: 10.3389/fphar.2022.821810 (PMC9213670; doi:10.3389/fphar.2022.821810)
Supplement: Supplementary file 1 [file DataSheet1.zip › Supplementary Material/Forest and Funnel Plots/Trigonella foenum-graecum/Fasting C-peptide.pdf]

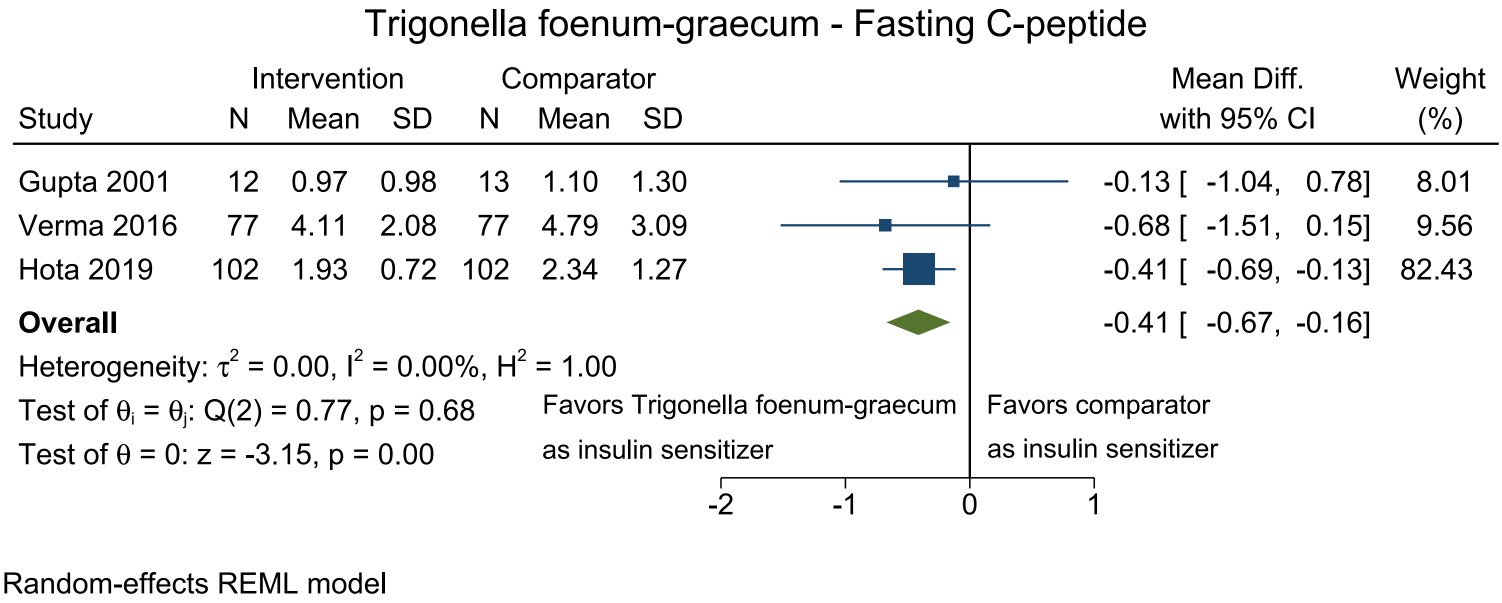
Trigonella foenum-graecum - Fasting C-peptide
Intervention
Comparator
Study
N
Mean
SD
N
Mean
SD
Mean Diff. with 95% CI
Weight (%)
Gupta 2001 12 0.97 0.98 13 1.10 1.30 -0.13 [-1.04, 0.78] 8.01
Verma 2016 77 4.11 2.08 77 4.79 3.09 -0.68 [-1.51, 0.15] 9.56
Hota 2019 102 1.93 0.72 102 2.34 1.27 -0.41 [-0.69, -0.13] 82.43
Overall -0.41 [-0.67, -0.16]
Heterogeneity: τ² = 0.00, I² = 0.00%, H² = 1.00
Test of θi = θj: Q(2) = 0.77, p = 0.68
Test of θ = 0: z = -3.15, p = 0.00
Favors Trigonella foenum-graecum as insulin sensitizer
Favors comparator as insulin sensitizer
-2
-1
0
1
Random-effects REML model
